# Auxin meets BR: OsIAA7 teams up with OsGSK2 to destabilize OsBZR1 for rice seed size control

**DOI:** 10.1093/plcell/koaf152

**Published:** 2025-06-12

**Authors:** Jiajun Wang

**Affiliations:** Assistant Features Editor, The Plant Cell, American Society of Plant Biologists; School of Life Sciences, Xiamen Key Laboratory of Plant Genetics, Xiamen University, Xiamen 361102, China

Rice is one of the most important food crops. With the rapid growth of the global population, increasing rice yield has become an urgent demand. Grain size is one of the key factors determining rice yield and quality; therefore, elucidating the genetic and molecular mechanisms regulating grain size is crucial for food security. Both auxin and brassinosteroid (BR) signaling pathways play pivotal roles in regulating grain size (Li et al. 2018). How these two signaling pathways interact and coordinate to regulate grain size remains unclear.

Auxin/indole-3-acetic acid (Aux/IAA) proteins are auxin co-receptors. In rice, there are 31 Aux/IAA members that can form diverse complexes with the Auxin Response Factor (ARF) DNA-binding transcription factors to regulate gene expression in various ways (Luo et al. 2018). Different AUX/IAA proteins may function through distinct signaling modules to regulate grain size. For example, OsIAA3 represses grain size by inhibiting OsARF25 (Zhang et al. 2018), while OsIAA10 promotes spikelet hull cell expansion and grain size by repressing OsARF4 (Ma et al. 2023). In new work, Ronghua Qiu and collaborators (Qiu et al. 2025) found that OsIAA7 enhances the interaction between SHAGGY-LIKE KINASE 2 (OsGSK2), a homolog of the BR signaling negative regulator in Arabidopsis BR INSENSITIVE 2 (BIN2), and its target, BRASSINAZOLE-RESISTANT 1 (OsBZR1), thereby promoting its phosphorylation and degradation, ultimately limiting grain size enlargement (Figure).

**Figure.**
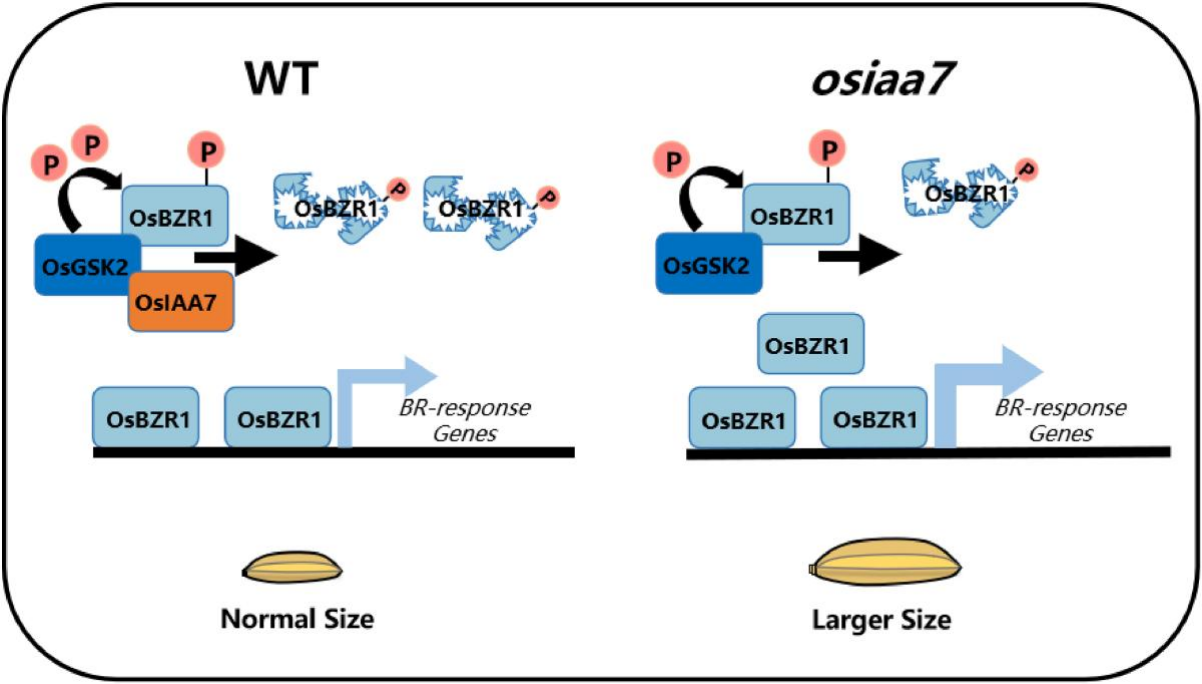
A proposed model illustrating how OsIAA7 regulates grain size via the OsGSK2–OsBZR1 module. In wild-type plants, OsIAA7 promotes OsGSK2-mediated phosphorylation of OsBZR1, leading to enhanced degradation of OsBZR1. Since OsGSK2 negatively regulates grain size while OsBZR1 acts as a positive regulator, this pathway fine-tunes grain development. In *osiaa7* knockout mutants, reduced phosphorylation of OsBZR1 by OsGSK2 results in accumulation of OsBZR1 protein, thereby shifting the balance toward larger grain size compared to wild-type plants. Reprinted from Qiu et al. (2025), Figure 8.

To identify auxin signaling genes involved in grain size regulation, the authors analyzed the expression pattern of *OsIAA7* using reverse transcription quantitative PCR and found that *OsIAA7* is highly expressed in the panicle. To further confirm this expression pattern, GUS staining was performed using *ProOsIAA7:GUS* transgenic plants, revealing that *OsIAA7* is expressed predominantly in the inflorescence, spikelets, and seeds. CRISPR/Cas9 knockout of *OsIAA7* resulted in *osiaa7* mutants with significantly increased grain length, width, and 1000-grain weight, while overexpression of *OsIAA7* did not significantly alter grain size or 1000-grain weight. Scanning electron microscopy observations revealed that the increased grain length in *osiaa7* mutants was primarily due to enhanced longitudinal cell elongation and division in the spikelet hull, while the increased grain width resulted from increased transverse cell elongation rather than cell division.

To further investigate the molecular mechanism by which OsIAA7 promotes grain size, the authors performed yeast 2-hybrid screening using a rice ZH11 cDNA library and found an interaction between OsIAA7 and OsGSK2, suggesting that *OsIAA7* may mediate crosstalk between auxin and BR signaling pathways. Using a variety of in vitro and in vivo techniques to study protein–protein interactions, the authors further validated the interaction between *OsIAA7* and *OsGSK2*. Yeast 2-hybrid assays with truncated OsIAA7 showed that OsGSK2 mainly interacts with core domains II and III of OsIAA7.

Unlike GSK3/SHAGGY-like kinase 41 (OsSK41/OsGSK5), which phosphorylates and degrades OsIAA10 to release OsARF4 (Ma et al. 2023), OsGSK2 does not phosphorylate or promote the degradation of OsIAA7. Interestingly, yeast 3-hybrid, pull-down, and Co-IP assays revealed that OsIAA7 promotes the interaction between OsGSK2 and OsBZR1. In vitro kinase assays showed that OsIAA7 enhances the phosphorylation of OsBZR1 by OsGSK2. Correspondingly, the phosphorylation level of endogenous OsBZR1 was significantly elevated in spikelets of *OsIAA7-HA* overexpression lines. Additionally, the authors found that while OsIAA7 does not affect the transcript levels of *OsBZR1*, the endogenous OsBZR1 protein levels were elevated in the flag leaves and spikelets of *osiaa7* CRISPR knockout mutants but significantly reduced in *OsIAA7-HA* overexpression lines. RNA-seq analysis showed that many yield-related regulatory genes were differentially expressed in the *osiaa7-2* mutant, and most of their promoters have OsBZR1-binding elements, suggesting that OsIAA7 may control grain size by modulating OsBZR1-mediated gene expression.

Moreover, genetic analyses confirmed that OsIAA7 is involved in the BR signaling pathway and functions upstream of OsGSK2. By examining lamina inclination angles, a common readout of BR signaling in rice, the authors found that *osiaa7* mutants exhibit increased sensitivity to exogenous 24-epibrassinolide treatment, while *OsIAA7* overexpression lines are insensitive. Grain size analysis showed that overexpression of *OsGSK2* in the *osiaa7-2* background significantly rescued the enlarged grain phenotype of *osiaa7-2*. Compared with the wild type, *osiaa7-2 Ubi:OsGSK2* exhibited shorter grain length, thinner grain thickness, a reduced length-to-width ratio, and decreased 1000-grain weight. In contrast, overexpression of *OsIAA7* in the *osgsk2-2* background did not significantly alter grain size. These data indicate that OsIAA7 acts upstream of OsGSK2 in genetic regulation, and that the *OsIAA7–OsGSK2* module plays a critical role in regulating grain size in rice.

In summary, the work by Qiu et al. (2025) demonstrates that OsIAA7 limits grain enlargement by enhancing the interaction between OsGSK2 and OsBZR1, thereby increasing OsBZR1 phosphorylation and promoting its degradation (Figure).

## Recent related articles in *The Plant Cell*

- Yu et al. (2024) found that the OsLAC-OsTTL module regulates grain yield in rice by modulating BR signaling through phosphorylation-dependent turnover of the BR repressor OsTTL.
- Li et al. (2025) revealed that DEP1 promotes BR signaling in rice by facilitating BR-induced nuclear entry and enhancing OsMYB86-mediated activation of *BU1*, which interacts with bHLH proteins to relieve IBH1-mediated repression of *OsARF11*, thereby linking G-protein signaling to BR–auxin crosstalk and regulating yield-related traits.
- Duan et al. (2023) reported that OsSHI1 functions as a central transcriptional hub that integrates auxin, BR, and abscisic acid signaling to coordinately regulate rice growth and stress responses.

## Dive Curated Terms

The following phenotypic, genotypic, and functional terms are of significance to the work described in this paper:

BIN2 Gramene: AT4G18710
BIN2 Araport: AT4G18710

OsIAA7 Gramene: LOC_Os02g13520
OsIAA7 Araport: LOC_Os02g13520

OsGSK2 Gramene: Os05g0207500
OsGSK2 Araport: Os05g0207500

OsARF11 Gramene: LOC_Os04g56850
OsARF11 Araport: LOC_Os04g56850

DEP1 Gramene: Os09g0441900
DEP1 Araport: Os09g0441900

OsIAA3 Gramene: LOC_Os01g13030
OsIAA3 Araport: LOC_Os01g13030

OsARF25 Gramene: LOC_Os12g41950
OsARF25 Araport: LOC_Os12g41950

OsIAA10 Gramene: LOC_Os02g57250
OsIAA10 Araport: LOC_Os02g57250

OsARF4 Gramene: LOC_Os01g70270
OsARF4 Araport: LOC_Os01g70270

OsBZR1 Gramene: LOC_Os07g39220
OsBZR1 Araport: LOC_Os07g39220
